# Using enhanced-mitophagy to measure autophagic flux

**DOI:** 10.1016/j.ymeth.2014.11.014

**Published:** 2015-03-15

**Authors:** Alice D. Baudot, Martina Haller, Michaela Mrschtik, Stephen W.G. Tait, Kevin M. Ryan

**Affiliations:** a Cancer Research UK Beatson Institute, Garscube Estate, Switchback Rd, Glasgow G61 1BD, UK; b Institute of Cancer Sciences, University of Glasgow, Garscube Estate, Switchback Rd, Glasgow G61 1BD, UK

**Keywords:** Macroautophagy, Autophagic flux, Mitophagy, Parkin

## Abstract

Macroautophagy (hereafter termed autophagy) is a cellular membrane-trafficking process that functions to deliver cytoplasmic constituents to lysosomes for degradation. Autophagy operates at basal levels to turn over damaged and misfolded proteins and it is the only process for the turnover of organelles. The process is therefore critically important for the preservation of cellular integrity and viability. Autophagy is also highly adaptable and the rate and cargoes of autophagy can be altered to bring about desired cellular responses to intracellular and environmental cues, disease states and a spectrum of pharmaceutical drugs. As a result, there is much interest in understanding the dynamics of autophagy in a variety of situations. To date, the majority of assays to monitor autophagy either measure changes in a parameter of the process at a set point in time or use markers/tracers to monitor flow of membrane-bound proteins from one point in the process to another. As such, these assays do not measure changes in endogenous cargo degradation which is the ultimate end-point of the autophagy process. We describe here an assay to measure autophagic cargo degradation by engineering cells to degrade mitochondria en masse. We show that this ‘enhanced-mitophagy’ assay can be used to measure differences in the rate of autophagy between different cells or in response to agents which are known to promote or inhibit autophagic flux. We consider therefore that this assay will prove to be a valuable resource for investigations in which autophagy is considered important and is believed to be modulated.

## 1 Introduction

Autophagy which literally means self-eating (auto – self; phagy – eating) is a group of cellular processes that deliver cytoplasmic cargoes to lysosomes for subsequent degradation. Three forms of autophagy have been described – macroautophagy, microautophagy and chaperone-mediated autophagy – which are individually characterized by the mechanism through which lysosomal delivery occurs [1]. Macroautophagy is the most extensively studied of these processes and for simplicity we will refer to it hereafter as autophagy.

Autophagy is initiated upon the formation of a structure called the isolation membrane which has been shown to originate from multiple locations within the cell [2–5]. Two ubiquitin-like mechanisms are then engaged to grow the membrane into a double-membraned ball termed an autophagosome, which is the characteristic organelle of this specific process. Upon autophagosome formation, two specific events must occur that are critical for cargo recruitment [6]. Firstly, as the membrane grows, it must incorporate a lipidated form of microtubule-associated protein light chain 3 (LC3). In cells with basal autophagy, the majority of LC3 exists as an un-lipidated soluble form termed LC3-I in the cytoplasm. Upon promotion of autophagy a greater proportion of LC3 becomes conjugated to phosphatidylethanolamine to form a version of the protein called LC3-II which integrates into the autophagosome membrane via the lipid moiety [7]. LC3-II serves both as a structural component of the autophagosome membrane as well as a critical factor involved in accrual of cargo.

The second important factor for cargo recruitment is the engagement of adaptor proteins. This group of proteins which includes p62/SQSTM1, NBR1, OPTN and BNIP3L (Nix) act as bridges between LC3 on the autophagosome membrane and cargoes marked for autophagic degradation [8]. The utilization of adapter proteins enables selectivity in autophagy. Various forms of selective autophagy have been described including pexophagy – the selective removal of peroxisomes and mitophagy – the selective removal of mitochondria.

Following autophagosome formation and cargo recruitment, membrane fusions can then occur between autophagosomes and endosomes/multivesicular bodies, but ultimately fusion occurs with a lysosome resulting in the formation of a new organelle, the autolysosome. In this organelle, the acidic hydrolases provided by the lysosome break down the cargo of the autophagosome into constituent parts which are then transferred into the cytoplasm where they are either catabolized further or recycled into biosynthetic pathways [1].

The study of autophagy has in recent years received considerable attention due to its involvement and/or perturbation in various forms of human disease [1]. In addition, because the dynamics of autophagy can be easily regulated, there is also a lot of interest in manipulating autophagy for therapeutic gain. For example, studies have shown that the promotion of autophagy can facilitate clearance of the aggregated proteins associated with neurodegenerative diseases such as Huntington’s and Parkinson’s disease [9]. Conversely, several studies have indicated that certain tumours can be dependent on autophagy indicating that autophagy inhibitors may be useful agents for cancer chemotherapy [10–12].

In order to understand and have the ability to manipulate autophagy in any given context, it is critical to be able to determine the rate of autophagic flux in normal versus diseased cells and before and after drug treatments. To date, the majority of studies on autophagy utilize Western blotting to measure LC3-II levels or immunofluorescence (IF) to detect the balance of LC3-I (diffuse staining) versus LC3-II (punctate staining indicative of autophagosomes). While this gives a measure of the number of autophagosomes within either individual cells (IF) or a cell population (Western blotting), making assessments regarding autophagic flux by these means is problematic. This is due to the fact that autophagosomes represent a transient mid-point in the entire autophagy process. As a result, increased numbers of LC3-positive puncta and/or increased LC3-II levels can represent an increase in autophagosome formation, but this can conversely also represent a blockage or impairment in autophagosome turnover following autophagosome formation [13]. Because of this limitation, a number of other assays have been developed to monitor flux between autophagosomes and lysosomes by monitoring for example, the traffic of fluorescent-tagged versions of LC3 which give different fluorescent signals at neutral pH (i.e., while in the autophagosome) versus the more acidic pH in the lysosome [14]. These assays yield a considerable amount of information when compared to analysis of steady-state levels of LC3. However, they are still limited in that they do not measure cargo digestion which is the ultimate endpoint of the autophagy process.

The ability to monitor cargo degradation during autophagy has been hampered by the current paucity of known specific cargos. As a surrogate, analysis of the degradation of adapter proteins such as p62/SQSTM1 is useful in some situations, but not in others, for instance in cases where p62/SQSTM1 is not utilized as an adaptor protein. In addition, the levels of p62/SQSTM1 have been shown to be modulated at the transcriptional level in response to changes in p62/SQSTM1 protein levels [15–17] – an event which clearly confounds to the ability to monitor loss of p62/SQSTM1 degradation by autophagy. Bearing in mind the issues regarding many current autophagy assays, we describe here the development of an assay to monitor the autophagic degradation of mitochondria which can be used to determine differences in the rate of autophagic cargo digestion between different cells or in response to external stimuli.

## 2 Materials and methods

### 2.1 Cell culture and treatments

All cell lines were cultured in DMEM (Gibco) containing 10% FBS, 4.5 g l^−1^ glucose, 1 mM l-glutamine, 0.11 g l^−1^ pyruvate and maintained at 37 °C in 5% CO_2_ atmosphere. For mitochondria depletion, cells were treated with 1 μM antimycin A (Sigma A8674) and 1 μM oligomycin (Sigma O4876) every 12 h for the indicated time periods as previously described [18]. We recommend that a titration of these drugs is undertaken in different cell systems in order to determine the most effective concentration to cause mitochondrial damage and depletion in any given system.

In order to activate autophagy, cells were treated with 100 nM rapamycin (LCL laboratories R-5000) or 150 μM of deferoxamine mesylate (DFO) for 2 h prior treatment with oligomycin and antimycin A. For the inhibition of the autophagic flux cells were treated with 10 μM hydroxychloroquine (Sigma) or 100 nM bafilomycin A1 (Sigma) together with antimycin A and oligomycin.

### 2.2 Retroviral infections

3T3-SA-YFP-Parkin and SVEC-YFP-Parkin were generated as previously described [19]. To generate Saos2-YFP-Parkin, Saos2 EcoR were retrovirally infected as previously described [20]. Briefly, Phoenix Eco packaging cells were transfected with pLZRS-YFP-Parkin by CaPO_4_ precipitation. Retroviral supernatants were added to Saos2-EcoR cells together with Polybrene (hexadimethrine bromide; Sigma). After the third round of infection cells were selected for 7 days in Zeocin (200 μg/ml, Invitrogen). To generate ATG7 knockout mouse embryonic fibroblasts (MEFs), immortalised ATG7^flox/flox^ MEFs were retrovirally infected with pBabe-puro-Cre or a retroviral vector as control. Cells were then selected in 2.5 μg/ml puromycin for 5 days. ATG7^flox/flox^ and ATG7^−/−^ were then retrovirally infected using pLZRS-YFP-Parkin as described above.

### 2.3 Western blotting

Following treatments, all floating cells were discarded and attached cells were lysed in buffer containing 1% triton-X, 0.1% SDS, 50 mM HEPES pH 7.5, 150 mM NaCl, 100 mM NaF, 10 mM EDTA, 10 mM Na_4_P_2_O_7_ and protease inhibitors (Roche). After protein concentration determination using BCA assay (Sigma), cell lysates were separated by SDS–PAGE and transferred onto Immobilon®-P membranes (Millipore). Membranes were probed with MitoProfile®Membrane Integrity WB Antibody cocktail (Abcam ab110414), anti-LC3B (Cell Signaling Technology 2775) and anti β-actin (Abcam ab8227) antibodies.

### 2.4 Statistical analysis

For time course studies, data were analyzed using a general linear model repeated ANOVA.

## 3 Theory

**Fig. 1:**
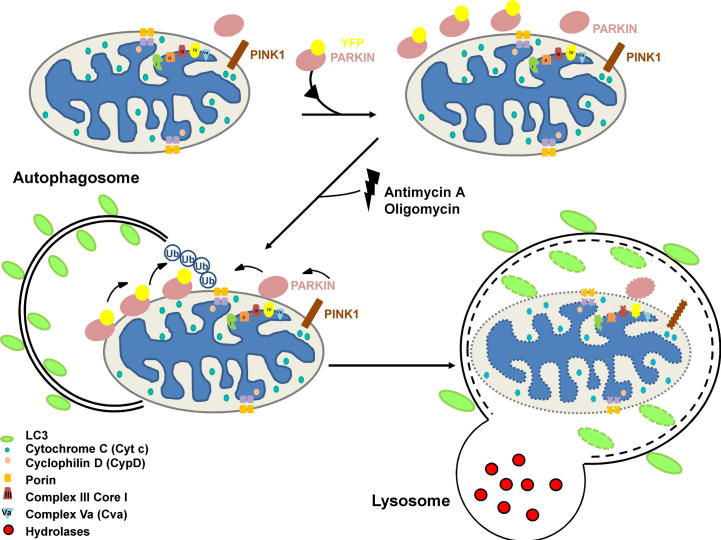
Parkin overexpression promotes mitophagy upon mitochondrial depolarization. In normal conditions the ser/thr kinase PINK1 is constitutively localized at the mitochondria. After overexpression of exogenous YFP-Parkin and treatment with antimycin A and oligomycin, mitochondrial proteins are depleted due to elimination of mitochondria en masse.

Parkin-dependent mitophagy involves PTEN-induced putative kinase 1 (PINK1) and a ubiquitin ligase complex containing Parkin (Fig. 1). Studies have shown that the ectopic expression of Parkin in cells followed by treatment with agents that perturb mitochondrial function results in the complete removal of mitochondria over time [21,19]. We reasoned that by providing the cell with an excess endogenous cargo (damaged mitochondria), then the rate of loss of mitochondria would be directly related to the autophagic rate/flux within the cell. As a result, we considered that that this process of ‘enhanced-mitophagy’ could be used as an assay to measure differences in autophagic flux between cells and in response to external stimuli.

## 4 Results

### 4.1 Expression of exogenous Parkin enables ‘enhanced-mitophagy’

**Fig. 2:**
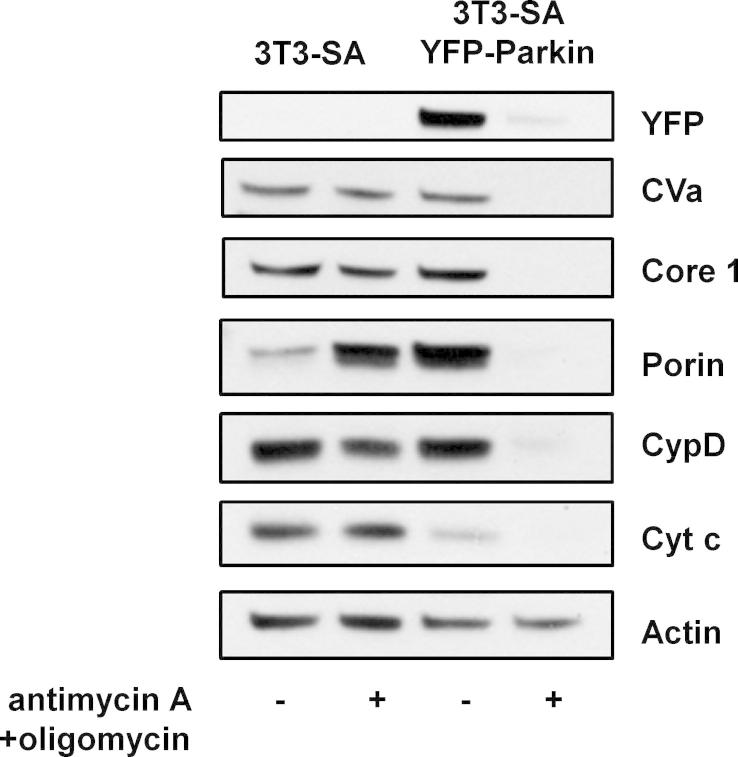
Expression of exogenous Parkin enables mitophagy. 3T3-SA cells with or without expression of YFP-Parkin were treated with 1 μM antimycin A + 1 μM oligomycin and analyzed by Western Blotting after 48 h using the MitoProfile Membrane integrity WB Antibody Cocktail. Actin was used as a loading control. The immunoblots shown are representative of what was seen in at least three independent experiments.

To test our hypothesis that enhanced-mitophagy could be utilized to measure autophagic flux, we employed a commercial panel of antibodies which enables Western blotting for a number of mitochondrial proteins at the same time. 3T3-SA cells which had been infected with retrovirus expressing Parkin fused to the yellow fluorescent protein (YFP-Parkin) and parental control cells were treated with antimycin A (an inhibitor of the electron transport chain) and oligomycin (an inhibitor of ATP synthase) for 2 days. Cell lysates were then prepared and examined by Western blotting. This revealed that over this time frame there was no noticeable depletion of mitochondrial proteins in control cells whereas all mitochondrial proteins were dramatically depleted in cells expressing exogenous YFP-Parkin (Fig. 2).

### 4.2 Enhanced-mitophagy can be used to reveal differences in autophagic flux between cells

**Fig. 3:**
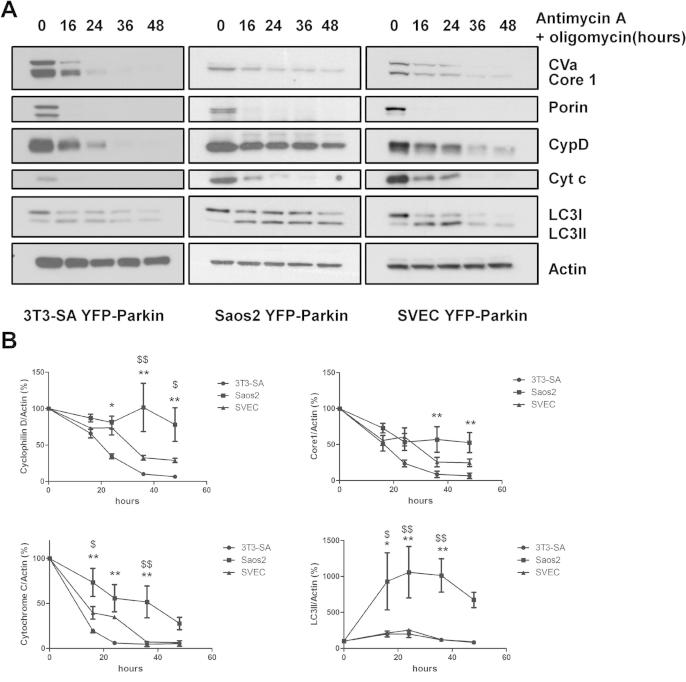
Induced-mitophagy can be used to measure differential autophagy kinetics in different cell lines. (A) 3T3-SA-YFP-Parkin, Saos2 YFP-Parkin and SVEC YFP-Parkin cells were treated with 1 μM antimycin A + 1 μM oligomycin for the indicated time periods. Cell lysates were analyzed by Western blot using the MitoProfile Membrane integrity WB Antibody Cocktail, and an anti-LC3B antibody. Actin was used as a loading control. (B) Protein quantification from three independent experiments was determined by ImageJ software using the intensity of the band and expressed as mitochondria protein to actin ratio (%). ^∗,∗∗^Indicate significant differences between 3T3-YFP-Parkin and Saos-2 YFP-Parkin (^∗^*p* < 0.05; ^∗∗^*p* < 0.001) and $ indicates significant difference between SVEC YFP-Parkin and Saos-2 YFP-Parkin. ^$^*p* < 0.05; ^$$^*p* < 0.01.

Since autophagy is the only cellular mechanism for mitochondrial removal, we were interested to know if enhanced-mitophagy could be used to reveal differences in autophagic flux between cells. Saos-2 osteosarcoma cells and SVEC endothelial cells were therefore infected with YFP-Parkin to enable mass elimination of mitochondria by autophagy and the depletion of mitochondrial proteins was measured in these and YFP-Parkin-expressing 3T3-SA cells following treatment with antimycin A and oligomycin. This revealed that not only were there significant differences in the rate of loss of mitochondrial proteins between different cell lines, but there were also differences in the rate of depletion of different mitochondrial proteins in each individual cell line Fig. 3A and B). Notably, cytochrome *c* and Porin (also known as VDAC1) are depleted very rapidly in SVEC and 3T3-SA after treatment with antimycin A and oligomycin (Fig. 3A and B). As a result, we did not consider them useful in determining differential rates of autophagy in these cells over this time frame.

By contrast to the rapid loss of cytochrome *c* and Porin, depletion of Complex Va (CVa), Complex III Core 1 (Core 1) and cyclophilin D occurred at a progressive rate following treatment with antimycin A and oligomycin that was differential between the three cell lines analyzed (Fig. 3A). Quantification of the rate of loss of Core 1 and cyclophilin D revealed that depletion was significantly more rapid in 3T3-SA and SVEC when compared to Saos-2 (Fig. 3B). This indicates a quicker loss of mitochondria in 3T3-SA and SVEC which we consider is due to differences in the autophagic rate between these cells and Saos-2. To address this point further, we also analyzed the relative accumulation of the classical autophagy markers LC3-II and p62 in the three cell lines following treatment with the autophagy inhibitor, chloroquine. This revealed that the accumulation of LC3-II and p62 was much slower in Saos-2 cells when compared with 3T3-SA and SVEC indicating that Saos-2 have a lower autophagic rate (Supplementary Fig. 1A and B). Since this result is completely in line with what we observed using enhanced-mitophagy, we conclude that by providing a large pool of endogenous substrate our assay measures differences in the general autophagic flux/capacity of the cell and does not simply reveal differences in mitophagy.

### 4.3 Enhanced-mitophagy can detect changes in autophagic flux caused by external stimuli or genetic loss of an essential autophagy gene

**Fig. 4:**
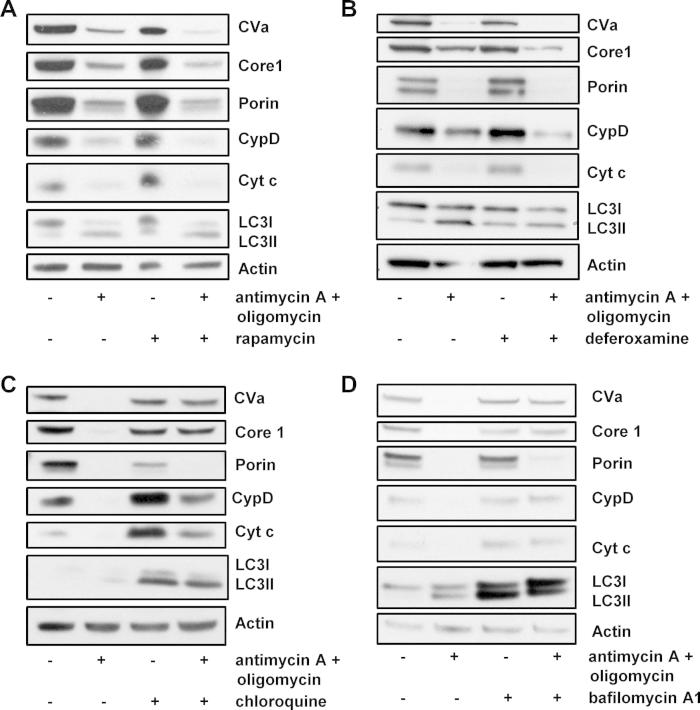
Promotion and inhibition of autophagy can be detected in cells expressing exogenous Parkin. (A) 3T3-SA cells expressing YFP-Parkin were treated with 1 μM antimycin A + 1 μM oligomycin with or without addition of 100 nM rapamycin as indicated to promote autophagy. After 16 h cells were analyzed for mitochondrial protein depletion by Western blotting. The immunoblots shown are representative of what was observed in three independent experiments. (B) Saos2-YFP Parkin cells were treated with 150 μM deferoxamine mesylate (DFO) for 1 h before adding 1 μM antimycin A + 1 μM oligomycin for 24 h. Lysates were then analyzed by Western blotting and are representative of the results observed on two separate occassions. (C and D) 3T3-SA YFP-Parkin cells were treated with 1 μM antimycin A + 1 μM oligomycin −/+ 10 μM chloroquine (C) or 100 nM bafilomycin (D) to inhibit autophagy. After 48 h, cell lysates were analyzed for mitochondrial protein depletion by Western blotting. Actin was used as a loading control. The immunoblots shown are representative of what was seen in three independent experiments.

To determine if loss of CVa, Core 1 and cyclophilin D following treatment with antimycin A and oligomycin was occurring by autophagy, we sought to determine if the loss of these proteins could be affected by agents that are known to affect autophagic flux. To this end, we firstly treated cells with rapamycin which is a well characterized inhibitor of mTOR Complex 1 and a potent inducer of autophagy (Supplementary Fig. 2A and B) [22] and with deferoxamine mesylate (DFO), a hypoxia mimetic and previously described inducer of autophagy [23]. Treatment with these drugs alone had little impact on the levels of mitochondrial proteins. However, in cells treated with rapamycin or DFO together with antimycin A and oligomycin there was a marked depletion of mitochondrial proteins that was greater than that observed in cells treated with antimycin A and oligomycin alone (Fig. 4A and B). The difference in loss of CVa, Core 1 and cyclophilin D caused by treatment was particularly clear, underscoring the usability of these proteins as markers of autophagic flux in these cells. A small difference was also seen for Porin in response to rapamycin, however, no change in cytochrome *c* levels was evident with either drug, ruling out its applicability in this assay.

We next assessed if the loss of mitochondrial proteins following treatment with antimycin A and oligomycin was affected by inhibition of autophagy. To do this we treated cells with the lysosomotropic agents hydroxychloroquine (Cq) or bafilomycin A1 (Baf A1) which raise the pH of the lysosome, thereby restricting the turnover stage of autophagy. As evidence of the effectiveness of these treatments, an accumulation of LC3-II was observed upon treatment with either drug (Fig. 4C and D). Treatment with Cq or Baf A1 also raised the levels of cytochrome *c* and reduced the levels of Porin (Fig. 4C and D). By contrast, the levels of CVa, Core 1 and cyclophilin D were largely unaffected (Fig. 4C and D).

The effect of Cq and Baf A1 on the loss of mitochondrial proteins upon exposure to antimycin A and oligomycin was also assessed. This revealed that the loss of CVa, Core 1 and cyclophilin D caused by antimycin A and oligomycin could be reversed by treatment with Cq and Baf A1 (Fig. 4C, D and Supplementary Fig. 3A, B). A statistically significant restoration of CVa, Core 1 and cyclophilin D levels was observed in response Cq (Fig. 4C and Supplementary Fig. 3A) and a statistically significant restoration of CVa and Core 1 was achieved by treatment with Baf A1 (Fig. 4D and Supplementary Fig. 3B). These data therefore confirm that CVa, Core 1 and cyclophilin D are indeed lost by autophagy upon treatment with antimycin A and oligomycin and that they can be used as reliable readouts of autophaghic flux in this method of enhanced-mitophagy.

**Fig. 5:**
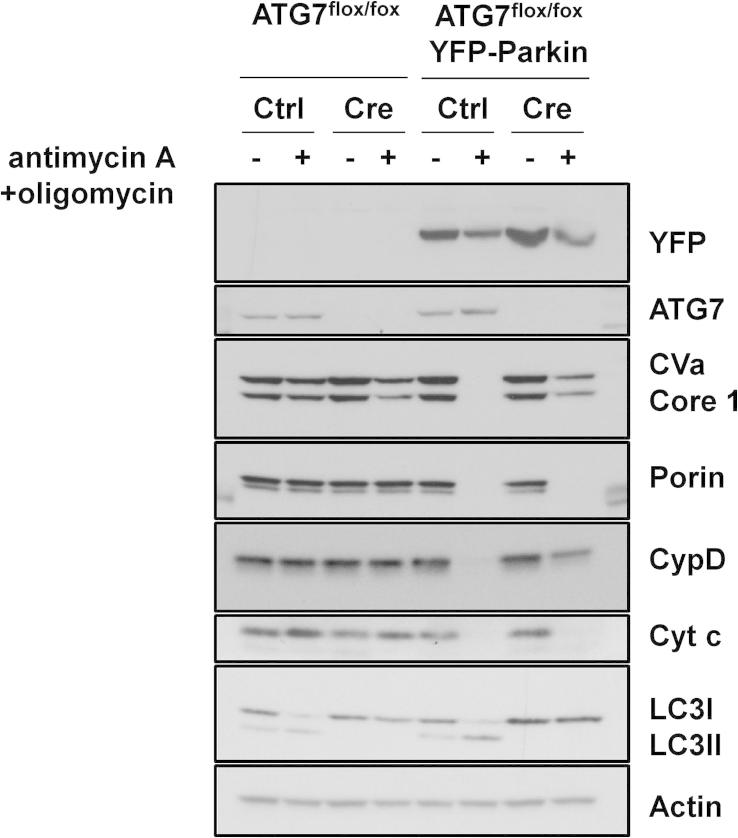
Enhanced-mitophagy can detect genetic loss of autophagy. ATG7^flox/flox^ MEFs expressing YFP-Parkin together with either a retrovirus expressing Cre recombinase or control virus were treated with 1 μM antimycin A + 1 μM oligomycin for 24 h. Lysates were analyzed by Western blotting was undertaken using the MitoProfile Membrane integrity WB Antibody Cocktail, an anti-LC3B antibody and an anti-actin antibody as a loading control. The immunoblots shown are representative of what was seen in three independent experiments.

We lastly decided to test if enhanced-mitophagy can detect genetic loss of autophagy. To do this we isolated fibroblasts from E13.5 mouse embryos which were homozygous for a floxed allele of the essential autophagy gene Atg7. Following isolation and immortalization, this floxed allele was recombined *in vitro* by infection with a retrovirus expressing Cre recombinase, with an ‘empty’ retroviral vector being used as control (Fig. 5). Subsequent infection of these cells with YFP-Parkin resulted in virtually complete ablation of CVA, Core 1, Porin, cyclophilin D and cytochrome *c* when control cells were treated with antimycin A and oligomycin (Fig. 5). By contrast, in YFP-Parkin-expressing Atg7-null cells while loss of Porin and cytochrome *c* occurs in a similar manner to what was observed in control cells, the depletion of CVa, Core 1 and cyclophilin D was markedly impaired due to loss of autophagy (Fig. 5). We therefore conclude that enhanced-mitophagy can be used to detect genetic loss of autophagy and that CVa, Core 1 and cyclophilin D are once again the reliable mitochondrial proteins to monitor when using this assay.

## 5 Concluding remarks

We describe here a method to analyze autophagic flux based on a system we have termed ‘enhanced-mitophagy’. We show that using this system we can detect differences in autophagic flux between different cell lines, in response to external stimuli and upon genetic loss of an essential autophagy gene. We believe this assay to be a true measure of the autophagic capacity of cells to degrade an endogenous cargo. As such, we consider it is much more informative than many current assays to measure autophagy. In this regard, it is notable that the changes we observed in the autophagic degradation of mitochondrial proteins is in line with what could be inferred regarding flux rates from LC3 and p62 Westerns in these cells. However, the analysis of LC3 by Western blotting alone cannot be extrapolated to make definitive statements regarding cargo digestion and p62 analysis is known to be hampered in some systems by changes in the expression as well as the degradation of the protein [15–17]. As a result, since analysis of LC3 and p62 has limitations, we believe that the enhanced-mitophagy assay has great value in complementing analysis of these proteins as markers of successful autophagy. It should be noted, however, that while we consider that enhanced-mitophagy can be used to assay the general autophagic flux/capacity of the cell and not just mitophagy, it cannot be used as a measure of the degradation rate of all potential autophagic cargoes. While it can be assumed that all cargoes are affected by the general autophagic flux/capacity of the cell, their degradation will for example, also be dependent on specific cues, protein modifications and the availability of sufficient amounts of any required adaptor proteins.

Perhaps one surprising outcome from this study was the realization that the depletion of different mitochondrial proteins was not equivalent upon treatment with antimycin A and oligomycin. We found that CVa, Core 1 and cyclophilin D were progressively depleted in a way that could reveal differences in autophagic rate between cells and that the levels of these proteins behaved as expected when autophagy was either promoted or inhibited. We therefore consider these proteins to be reliable readouts of autophagic flux when using this enhanced-mitophagy assay in these cells. By contrast, the levels of Porin and cytochrome *c* underwent changes following treatment with antimycin A and oligomycin that cannot be explained solely by changes in autophagic flux. For example, Porin levels decreased upon treatment with Cq and cytochrome *c* levels increased in this context without an equivalent increase in other mitochondrial proteins such as CVa and Core 1. It is easy to rationalize that cytochrome *c* may be affected in a manner independent of the absolute levels of mitochondria since for example, it is well established that cytochrome *c* can be released from mitochondria in response to certain apoptotic stimuli [24]. Changes in Porin levels, are though less easy to explain since it is an integral membrane protein in the outer mitochondrial membrane. It has, however, previously been shown that Tom20 – another mitochondrial outer membrane protein – is ubiquitylated by Parkin and turned over in the proteasome [25]. Since Porin is also directly ubiquitylated by Parkin it may also be possible that it is targeted for degradation in a manner independent of mitophagy [26]. This is undoubtedly worthy of further investigation in future studies. For the purposes of this study, however, from what we have observed we do not consider Porin and cytochrome *c* to be reliable markers of autophagic flux in the cells analyzed. As a result, we would advocate caution in the analysis of these proteins when using this enhanced-mitophagy assay in other systems. We would also like to make clear why we used antimycin A and oligomycin in our study and not the more conventional mitochondrial uncoupler carbonyl cyanide m-chlorophenylhydrazone (CCCP). This decision was made in light of previous studies which have shown that CCCP can interfere with the degradation of autophagic cargoes in the lysosome [27]. As a result, we do not recommend using CCCP in the enhanced-mitophagy assay.

Finally, while we conclude from this study that enhanced-mitophagy is a reliable assay of autophagic flux in 3T3-SA, Saos-2 and SVEC cells, we would also argue that this assay would be broadly applicable in multiple situations. Using the retroviral construct that we describe here, we believe that the assay could be utilized in multiple cell culture systems. In addition, the production of a YFP-Parkin lentivirus should also permit application of this assay in non-dividing cells, for example in senescent cell cultures or in the analysis of non-dividing tissue *ex vivo*. Moreover, through the utilization of different fluorescent tags (YFP or otherwise), it may also be possible to measure changes in autophagic flux *in vivo*, for example following drug treatment of animals carrying tumor xenografts which have been engineered to express a fluorescent-tagged Parkin and a fluorescent-tagged mitochondrial protein such CVa or cyclophilin D. We hope therefore that the findings we describe in this study act as a starting point for future studies designed to analyze autophagic flux in multiple experimental systems and in a variety of situations in which autophagy is considered important.

## Appendix A Supplementary data

Supplementary Fig. 1 3T3-SA-YFP-Parkin, Saos2 YFP-Parkin and SVEC YFP-Parkin cells were treated with 10 μM chloroquine for the indicated time periods. LC3 lipidation (A) and p62 accumulation (B) were assessed by Western blotting. Actin was used as a loading control. The immunoblots shown are representative of what was seen in three independent experiments.

Supplementary Fig. 2 (A) 3T3-SA YFP-Parkin were treated with 100 nM rapamycin for the indicated time periods and LC3 lipidation was assessed by Western blotting. Actin was used a loading control. (B) 3T3-SA YFP-Parkin were treated with 1 μM antimycin A + 1 μM oligomycin with or without addition of 100 nM rapamycin for 48 h and p70S6Kinase phosphorylation was assessed by Western blotting using an anti-Phospho p70s6Kinase Thr389 antibody. Loading was assessed using antibodies directed against total p70S6Kinase and actin.

Supplementary Fig. 3 Protein quantification from Fig. 4C and D was determined from three independent experiments using ImageJ software and expressed as the decrease in protein level following uncoupler treatment (%). *t*-Test (unpaired, two-tailed, assuming equal variances) was used for statistical analysis (^∗^*p* < 0.05; ^∗∗^*p* < 0.001).
