# Supplementary figures and images for: Using enhanced-mitophagy to measure autophagic flux
Source: Methods. 2015 Mar 15;75:105–11. doi: 10.1016/j.ymeth.2014.11.014 (PMC4358839; doi:10.1016/j.ymeth.2014.11.014)

## Supplementary figure 1

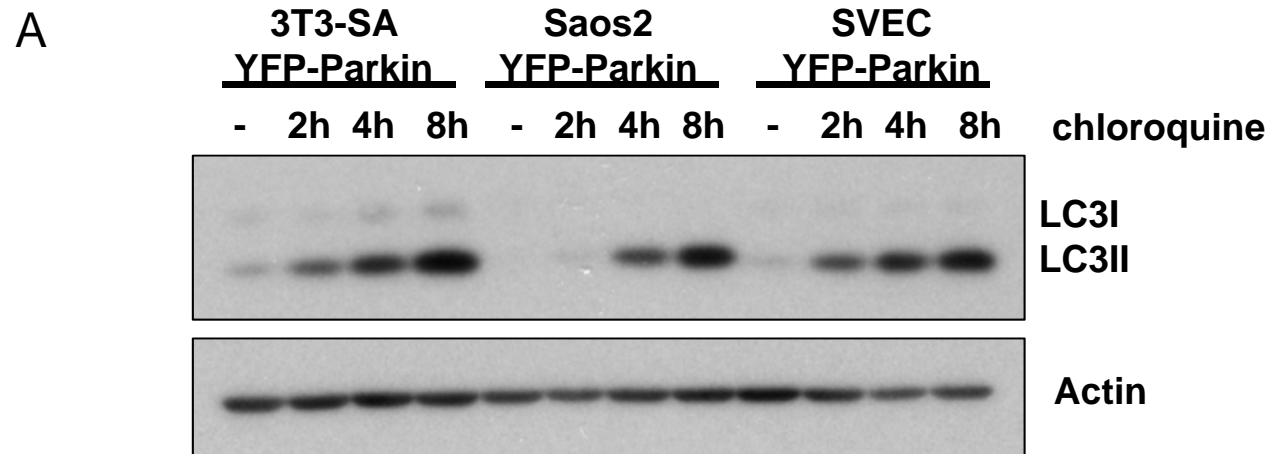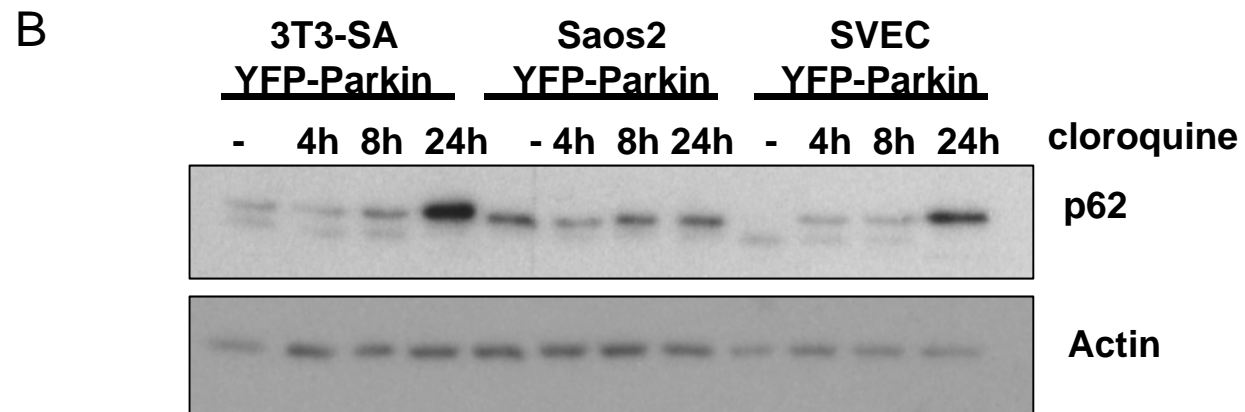

Supplement: Supplementary Fig. 1 — 3T3-SA-YFP-Parkin, Saos2 YFP-Parkin and SVEC YFP-Parkin cells were treated with 10 μM chloroquine for the indicated time periods. LC3 lipidation (A) and p62 accumulation (B) were assessed by Western blotting. Actin was used as a loading control. The immunoblots shown are representative of what was seen in three independent experiments. [file mmc1.pdf]

## Supplementary figure 2

A

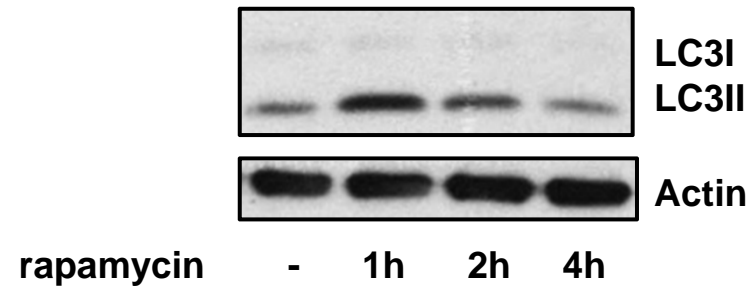

B

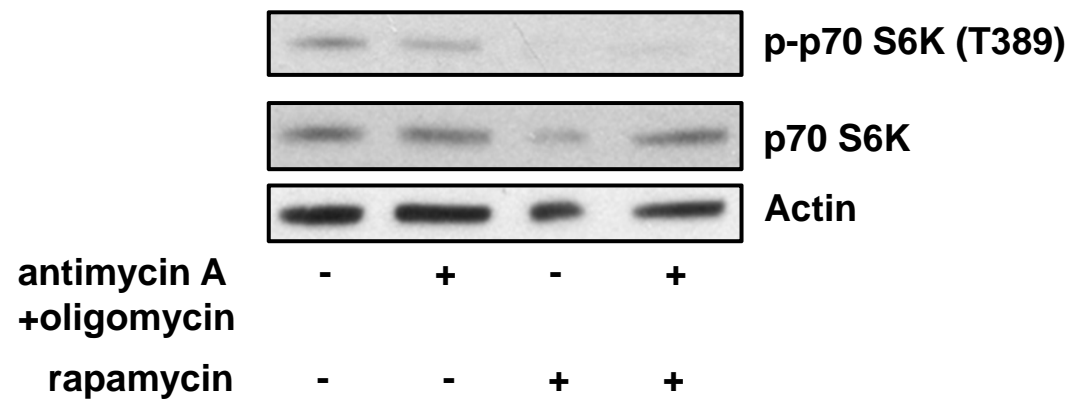

Supplement: Supplementary Fig. 2 — (A) 3T3-SA YFP-Parkin were treated with 100 nM rapamycin for the indicated time periods and LC3 lipidation was assessed by Western blotting. Actin was used a loading control. (B) 3T3-SA YFP-Parkin were treated with 1 μM antimycin A + 1 μM oligomycin with or without addition of 100 nM rapamycin for 48 h and p70S6Kinase phosphorylation was assessed by Western blotting using an anti-Phospho p70s6Kinase Thr389 antibody. Loading was assessed using antibodies directed against total p70S6Kinase and actin. [file mmc2.pdf]

## Supplementary figure 3

A

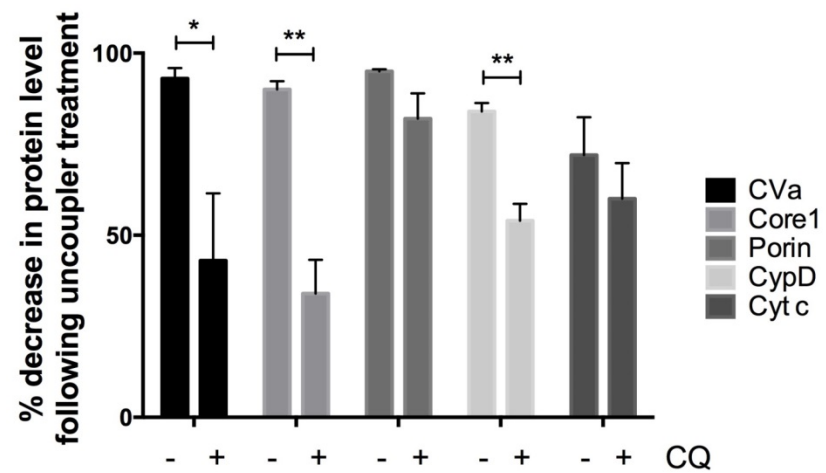

B

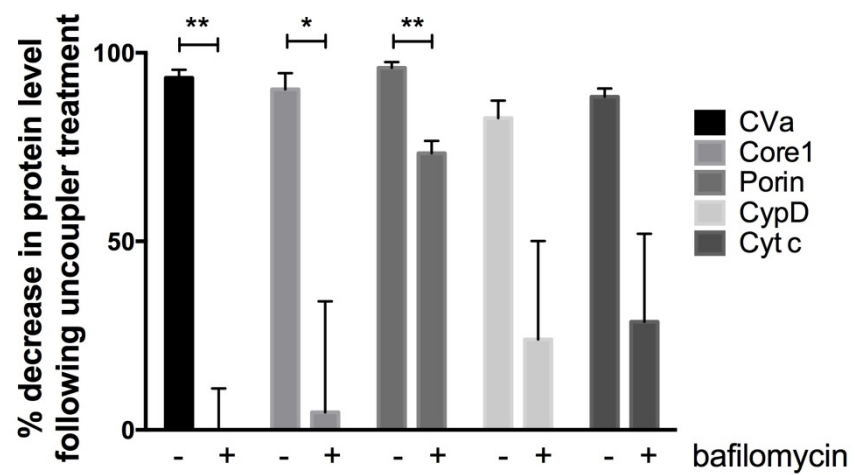

Supplement: Supplementary Fig. 3 — Protein quantification from Fig. 4C and D was determined from three independent experiments using ImageJ software and expressed as the decrease in protein level following uncoupler treatment (%). t-Test (unpaired, two-tailed, assuming equal variances) was used for statistical analysis (∗p < 0.05; ∗∗p < 0.001). [file mmc3.pdf]
